# Decorin-evoked paternally expressed gene 3 (PEG3) is an upstream regulator of the transcription factor EB (TFEB) in endothelial cell autophagy

**DOI:** 10.1074/jbc.M116.769950

**Published:** 2017-08-10

**Authors:** Thomas Neill, Catherine Sharpe, Rick T. Owens, Renato V. Iozzo

**Affiliations:** From the ‡ Department of Pathology, Anatomy, and Cell Biology and the Cancer Cell Biology and Signaling Program, Sidney Kimmel Medical College at Thomas Jefferson University, Philadelphia, Pennsylvania 19107 and; § LifeCell Corporation, Branchburg, New Jersey 08876

**Keywords:** autophagy, decorin, endothelial cell, proteoglycan, VEGF

## Abstract

Macroautophagy is a fundamental and evolutionarily conserved catabolic process that eradicates damaged and aging macromolecules and organelles in eukaryotic cells. Decorin, an archetypical small leucine-rich proteoglycan, initiates a protracted autophagic program downstream of VEGF receptor 2 (VEGFR2) signaling that requires paternally expressed gene 3 (PEG3). We have discovered that PEG3 is an upstream transcriptional regulator of transcription factor EB (TFEB), a master transcription factor of lysosomal biogenesis, for decorin-evoked endothelial cell autophagy. We found a functional requirement of PEG3 for TFEB transcriptional induction and nuclear translocation in human umbilical vein endothelial and PAER2 cells. Mechanistically, inhibiting VEGFR2 or AMP-activated protein kinase (AMPK), a major decorin-activated energy sensor kinase, prevented decorin-evoked TFEB induction and nuclear localization. In conclusion, our findings indicate a non-canonical (nutrient- and energy-independent) mechanism underlying the pro-autophagic bioactivity of decorin via PEG3 and TFEB.

## Introduction

The extracellular matrix is becoming a prominent and encompassing field of research; protein/protein interactions and enzymatic processing often lead to profound effects on the embedded cells and tissues (1–6). An emerging paradigm for extracellular matrix constituents, predominantly represented by soluble proteoglycans (7, 8), is autophagic regulation (9). Decorin, a prototypical member of the small leucine-rich proteoglycan gene family (10), directly interacts with a diverse set of receptor tyrosine kinases as a partial agonist for various biological activities (11–13), culminating in anti-oncogenic and angiostatic responses (14–17), both *in vitro* (18, 19) and *in vivo* (20–22).

High-resolution transcriptomics following systemic administration of decorin in triple-negative breast carcinoma orthotopic xenografts revealed differential gene expression exclusively within the tumor stroma (22). Among the subset of decorin-inducible genes was a genomically imprinted transcription factor of the Krüppel zinc finger family known as paternally expressed gene 3 (PEG3)^3^ (23, 24). PEG3 is a tumor suppressor (25, 26) whose expression is commonly lost because of promoter methylation (27, 28) or loss of heterozygosity (29).

We focused on PEG3 as both decorin and PEG3 disrupt Wnt signaling in a non-canonical way, independent of GSK3β (18, 30). During the course of these studies, we discovered that PEG3 was directly involved in regulating endothelial cell autophagy following exposure to either soluble decorin proteoglycan or its protein core (31, 32). Silencing PEG3 prevented induction of Beclin 1 and LC3 (31), two key components of the autophagic machinery (33). Moreover, PEG3 was required for maintaining basal levels of Beclin 1. Mechanistically, decorin requires the tyrosine kinase activity of vascular endothelial growth factor receptor 2 (VEGFR2), the dominant receptor tyrosine kinase expressed by endothelial cells (31).

Decorin modulates the phosphorylation of critical rheostatic kinases (AMPK and mammalian target of rapamycin (mTOR)) for maintaining the proper cellular balance of autophagy (34–37). Indeed, AMPK and mTOR (a primary component of mTORC1) play opposing roles in autophagic regulation, as AMPK is required for the initiation of autophagy via ULK1 phosphorylation (37–40) and mTOR for autophagic inhibition and termination (41, 42). We found sustained activation of the AMPKα catalytic subunit with concurrent suppression of mTOR signaling in endothelial cells (34). Notably, decorin-evoked autophagy occurs under nutrient-rich conditions, designating decorin as a non-canonical stimulus for autophagic induction.

The biosynthesis of new lysosomes is critical for achieving the objective of cargo degradation and nutrient recycling via the formation of terminal autophagolysosomes (43). Further, prolonged (or, in the case of decorin, excessive) autophagy depends on stable transcriptional programs capable of supporting long-term autophagic processes (44–46). Therefore, we focused on transcription factor EB (TFEB), a master regulator of lysosomal biogenesis with direct links to autophagic progression (47–51). Under anabolic conditions, activated mTORC1 directly phosphorylates TFEB, tethering it (in an inactive configuration) at the lysosomal surface via interactions with 14-3-3 proteins (52). This posits mTOR as a central regulator of TFEB function (43, 53, 54). Following autophagic stimulation or stress responses, TFEB is dephosphorylated by calcineurin and translocates to the nucleus for lysosomal gene expression by targeting a subset of genes collectively known as the Coordinated Lysosomal Expression and Regulation (CLEAR) network (47, 48).

As decorin suppresses mTOR activity and initiates prolonged autophagic responses, we evaluated the existence of a mechanistic link between PEG3 and TFEB for endothelial cell autophagy. We found that PEG3 is required for TFEB induction and nuclear translocation in a VEGFR2- and AMPK-dependent manner for decorin-evoked autophagy.

## Results

### Decorin-evoked PEG3 is required for TFEB induction

**Figure 1.**
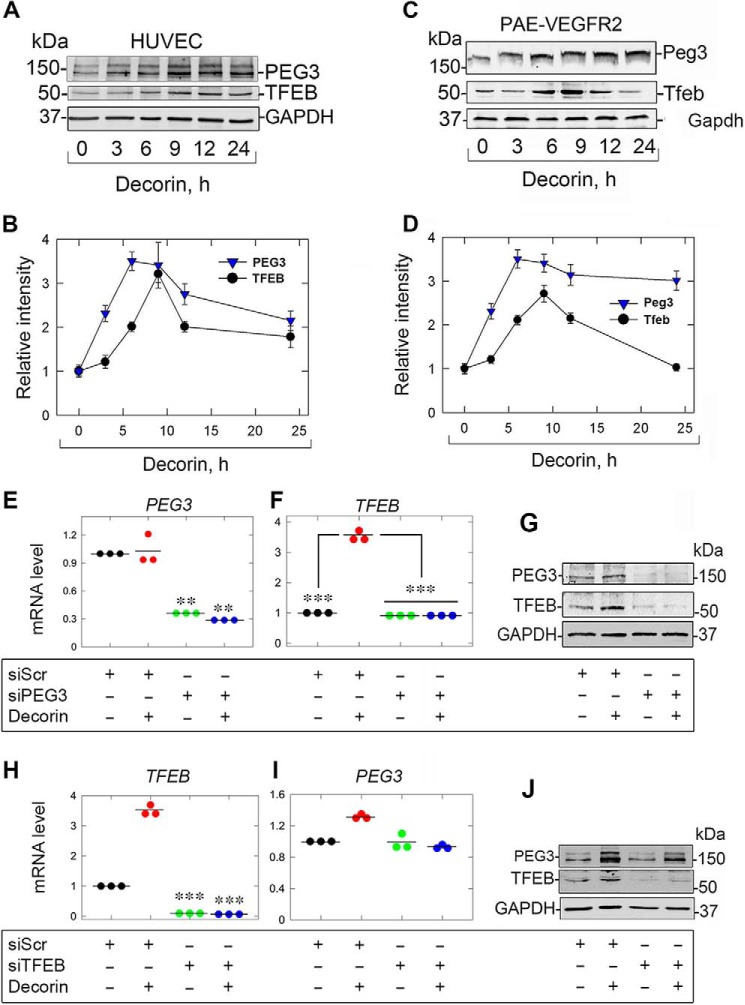
**Decorin evokes TFEB in a PEG3-dependent manner.** *A* and *B*, immunoblot and quantification of PEG3 and TFEB in HUVECs treated with decorin over time. *C* and *D*, identical experiment as in *A* and *B* in PAER2 cells. *E*, *PEG3* knockdown in the presence of decorin in combination with scramble siRNA (siScr) or siPEG3. *F*, *TFEB* analysis as in *E. G*, PEG3 and TFEB following PEG3 knockdown and challenge with decorin. *H*, identical RNAi verification experiment as in *E* but for *TFEB. I*, similar experiment as in *F* but for *PEG3. J*, similar experiment as in *G* but in the presence of siScr or siTFEB following decorin. GAPDH served as an internal loading control in *A*, *C*, *G*, and *J*. Gene expression in *E*, *F*, *H*, and *I* was normalized to *ACTB*. For the immunoblots in *A*, *C*, *G*, and *J* and quantifications in *B* and *D*, data are representative of three independent biological replicates each for PAER2 cells or HUVECs. The data in *E*, *F*, *H*, and *I* represent three independent biological replicates each for PAER2 cells or HUVECs. Statistical analyses were done via one-way ANOVA. **, *p* < 0.01; ***p < 0.001.

To evaluate a potential mechanistic link between PEG3 and TFEB, we conducted time course experiments in both human umbilical vein endothelial cells (HUVECs) and porcine aortic endothelial cells overexpressing VEGFR2 (PAER2). We found that PEG3 levels increased earlier and at a faster rate than TFEB induction at the same time points (Fig. 1, *A* and *C*). In HUVECs, following 9 h of decorin treatment, both PEG3 and TFEB levels were maximal and began to decline but remained high even at 24 h (Fig. 1, *A* and *B*). Further, the relative kinetics of Peg3 and Tfeb in PAER2 displayed a similar profile (Fig. 1*C*) insofar as Peg3 increased first, followed by a more gradual induction of Tfeb over time. However, unlike in HUVECs, Tfeb levels declined to baseline after reaching peak levels (at 9 h) whereas Peg3 remained elevated (Fig. 1*D*). We note that no differences in bioactivity were found between decorin and decorin core in this study (data not shown).

As the kinetics showed that PEG3 levels preceded those of TFEB, we evaluated the functional requirement of PEG3 for TFEB induction. After verification of PEG3 depletion (Fig. 1*E*), we found that TFEB was no longer induced following decorin treatment at either the mRNA (Fig. 1*F*) or protein (Fig. 1*G*) levels. Intriguingly, we found that loss of PEG3 alone was sufficient for decreasing basal TFEB protein (Fig. 1*G*) but not mRNA. These results are similar to the effect of PEG3 loss on Beclin 1 (31). In reciprocal experiments where TFEB was silenced (Fig. 1*H*), we found no significant change in *PEG3* mRNA (Fig. 1*I*). These data suggest that TFEB is downstream of PEG3. In accordance with our previous studies (31), we found that decorin does not evoke *PEG3* expression in endothelial cells (Fig. 1, *E* and *I*). In contrast, endorepellin, a matrix-derived molecule capable of inducing endothelial cell autophagy via VEGFR2, evokes *PEG3* mRNA levels (55–57). Finally, loss of TFEB did not abrogate decorin-evoked PEG3 protein levels (Fig. 1*J*) and had no appreciable effect on basal levels of PEG3 mRNA or protein (Fig. 1, *I* and *J*).

### De novo expression of Peg3 promotes Tfeb induction

**Figure 2.**
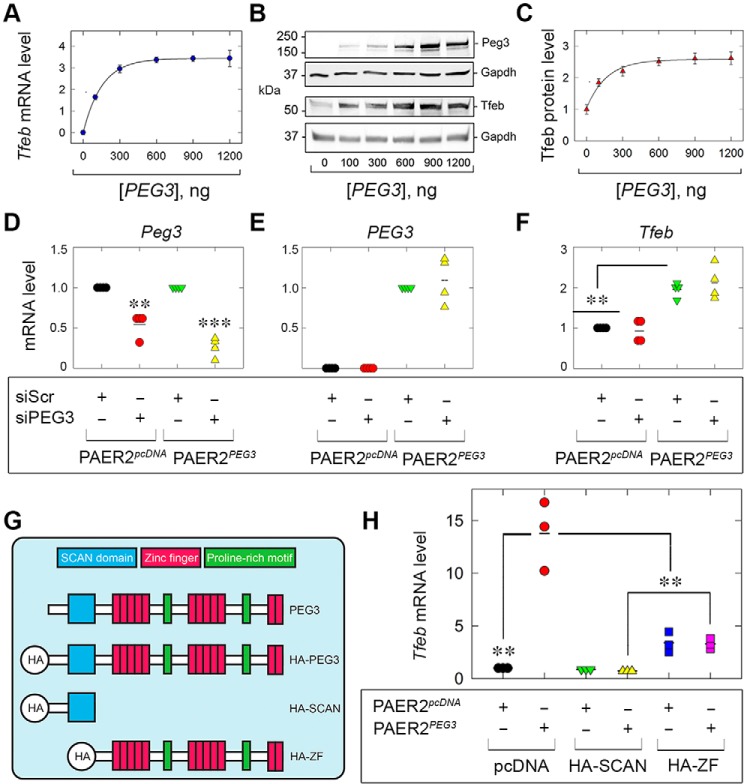
**Exogenous PEG3 drives TFEB.** *A*, *Tfeb* following increasing amounts of transfected *PEG3. B* and *C*, immunoblots (*B*) and quantification (*C*) for PEG3 and endogenous Tfeb. *D*, *E*, and *F*, analyses of endogenous *Sus scrofa Peg3* (*D*), *H. sapiens PEG3* (*E*), or *Sus scrofa Tfeb* (*F*) following supertransfection of either siScr or siPEG3 in control PAER2 cells (PAER2*^p^*cDNA) or stably expressing PEG3 PAER2 cells (PAER2*^PEG3^*) cells. *G*, schematic depicting known domains of full-length PEG3 and resulting HA-tagged truncations. *H*, expression analysis of *Tfeb* following supertransfection of HA-SCAN or HA-ZF into PAER2*^p^*cDNA or PAER2*^Peg3^* cells. The gene expression analyses in *A*, *D*, *E*, *F*, and *H* were normalized to *ACTB*. Gapdh served as an internal loading control in *B.* The quantifications in *C* are representative of three independent biological replicates in PAER2 cells. The data in *A*, *D*, *E*, *F*, and *H* represent three independent biological replicates in PAER2 cells. Statistical analyses were done via one-way ANOVA. **, *p* < 0.01; ***, *p* < 0.001.

Having established a role for PEG3 in regulating TFEB expression via loss-of-function experiments, we next ascertained the ability of PEG3 alone to drive TFEB. To this end, we transiently transfected PAER2 cells with HA-tagged human full-length PEG3 and evaluated porcine Tfeb. Following validation of increasing amounts of Peg3 via qPCR (data not shown), we found a dose-dependent increase in *Tfeb* mRNA that reached maximal output in as little as 300 ng (Fig. 2*A*) and maintained saturation for up to 1.2 μg of transfected *PEG3* (Fig. 2*A*).

Next, immunoblot analyses showed that increasing amounts of Peg3 promoted a significant increase in Tfeb starting with as little as 100 ng of Peg3 (Fig. 2, *B* and *C*). Notably, the profile obtained for Tfeb protein (Fig. 2*B*) paralleled that obtained for the mRNA (Fig. 2*A*). Collectively, these data reinforce the concept that Peg3 is necessary and sufficient for driving *Tfeb* expression and further substantiate the role of Peg3 in autophagic progression.

We further corroborated the mRNA and protein data gained via RNAi by generating PAER2 cells stably expressing human PEG3 (denoted as PAER2*^PEG3^*) and empty vector–expressing cells (PAER2*^p^*cDNA). This expression construct lacks the 3′ UTR (which harbors the siRNA recognition sites), rendering PEG3 siRNA-resistant. As we found that PEG3 is required to maintain basal TFEB (Fig. 1*G*), and *de novo* PEG3 is sufficient to drive Tfeb mRNA and protein (*cf.* Fig. 2, *A–C*), we supertransfected the stable PEG3-expressing cells and evaluated whether siRNA-resistant PEG3 could rescue *Tfeb* expression. Given the close sequence homology between humans and pigs, endogenous *Sus scrofa Peg3* was significantly reduced (Fig. 2*D*), but exogenous expression of *Homo sapiens PEG3* remained unperturbed (Fig. 2*E*). Evaluation of *Tfeb* demonstrated that stable expression, analogous to transient expression, of *PEG3* alone drove *Tfeb* (Fig. 2*F*). Importantly, despite transfection of a *PEG3*-specific siRNA, the presence of the siRNA-resistant PEG3 maintained significantly induced *Tfeb* mRNA (Fig. 2*F*).

Structurally, PEG3 is composed of an N-terminal SCAN domain required for protein–protein interactions and an extended C terminus of 12 C_2_H_2_ Krüppel-like zinc fingers (interspersed with proline-rich regions) for DNA binding and transcriptional regulation (Fig. 2*G*) (23, 58, 59). Generating HA-tagged truncation fragments (Fig. 2*G*) of either the SCAN domain (HA-SCAN) or the zinc fingers (HA-ZF) prevented nuclear translocation of these fragments following decorin stimulation in comparison with full-length HA-tagged PEG3 (60). We therefore expressed these fragments in our PAER2*^PEG3^* cells and assayed *Tfeb* expression to determine whether they acted in a dominant negative fashion. Surprisingly, HA-SCAN completely blocked PEG3-driven *Tfeb* expression compared with empty vector–transfected PAER2*^PEG3^* cells (Fig. 2*H*). Introduction of HA-ZF resulted in significantly abrogated Tfeb levels (Fig. 2*H*). Interestingly, transfection of HA-ZF alone in PAER2*^p^*cDNA was sufficient to drive *Tfeb* mRNA, suggesting a posttranscriptional pathway, as HA-ZF does not enter the nucleus (60). Collectively, these data establish that PEG3 is required for decorin-mediated TFEB induction and place PEG3 upstream of TFEB.

### TFEB induction relies on VEGFR2 and AMPK signaling

**Figure 3.**
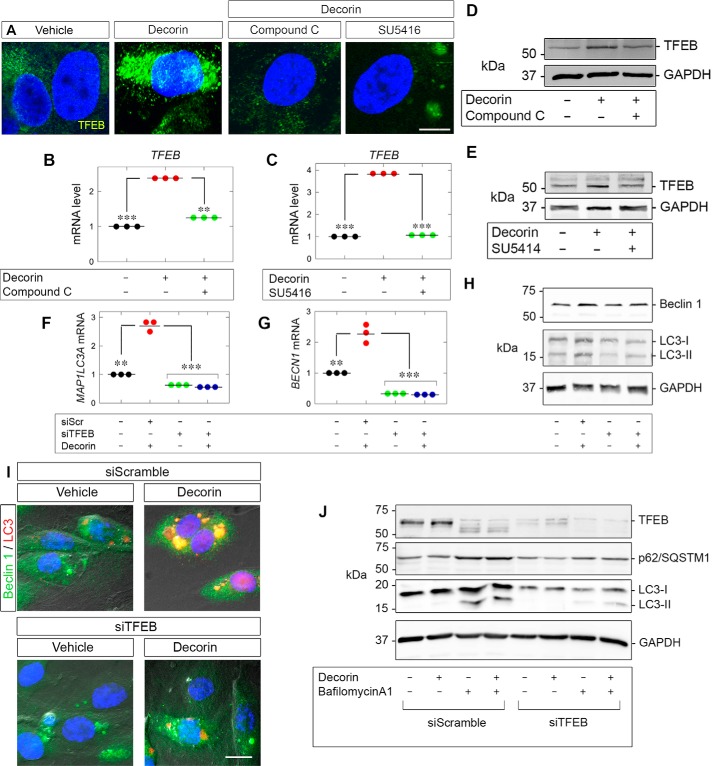
**Decorin evokes TFEB via VEGFR2/AMPK.** *A*, confocal microscopy depicting TFEB (*green*) following decorin (6 h) alone or in combination with Compound C (30 μm) or SU5416 (30 μm) in HUVECs. Nuclei (*blue*) were visualized with DAPI. *Scale bar* = 10 μm. *B* and *C*, *TFEB* with decorin in combination with Compound C (*B*) or with SU5416 (*C*). *D* and *E*, TFEB with decorin in combination (Compound C (*D*) or SU5416 (*E*)). *F* and *G*, *BECN1* (*F*) or *MAP1LC3A* (*G*) following *TFEB* knockdown (the same authenticated RNA samples as used in Fig. 1*H*). *H*, immunoblots depicting Beclin 1 and LC3 on the same samples as authenticated in Fig. 1*J. I*, DIC microscopy of autophagosomes stained for Beclin 1 (*green*) and LC3 (*red*) following transfection of siScr or siTFEB in conjunction with decorin (6 h). Nuclei (*blue*) were visualized with DAPI. *Scale bar* = 10 μm. *J*, PAER2 cells transiently transfected with siScr or siTFEB and treated with Bafilomycin A1 (100 nm) and/or decorin (6 h). GAPDH served as an internal loading control in *D*, *E*, *H*, and *J*. The gene expression analyses in *B*, *C*, *F*, and *G* were normalized to *ACTB*. For confocal microcopy in *A* and DIC in *I*, at least five and ten fields per condition, respectively, were captured for each of three biological replicates in HUVECs. For the immunoblots in *D*, *E*, *H*, and *J*, data are representative of at least three independent biological replicates in HUVECs or PAER2 cells. The data *B*, *C*, *F*, and *G* represent at least three independent biological replicates in HUVECs. Statistical analyses were done via one-way ANOVA. **, *p* < 0.01; ***, *p* < 0.001.

Endothelial cell autophagy in response to soluble decorin proceeds in a VEGFR2/AMPK-dependent manner (31, 34). To address the role of AMPK, we utilized Compound C (dorsomorphin), a potent and reversible ATP-competitive inhibitor of AMPK that directly interacts with the catalytic α subunit (37). Compound C significantly abolished decorin-evoked TFEB induction (Fig. 3*A*). Moreover, blocking the kinase activity of VEGFR2 with SU5416 also prevented an increase in TFEB (Fig. 3*A*). In both cases, TFEB was excluded from the nuclear compartment when the kinase activity of either AMPK or VEGFR2 was abrogated (Fig. 3*A*).

We then tested whether the transcriptional induction of *TFEB* was dependent on AMPK and VEGFR2. Blocking either AMPK (Fig. 3*B*) or VEGFR2 (Fig. 3*C*) significantly prevented decorin-evoked *TFEB* expression. These results were extended at the protein level insofar as Compound C (Fig. 3*D*) or SU5416 (Fig. 3*E*) precluded an increase in TFEB following decorin stimulation. We conclude that decorin requires the VEGFR2–AMPK signaling axis for proficient nuclear translocation of TFEB protein and proper induction of TFEB.

### Autophagosome formation and autophagic flux require TFEB

A hallmark of autophagy is the induction of Beclin 1 and conversion of LC3-I to its phosphatidylethanolamine-conjugated form, LC3-II, coincident with the formation of dually positive Beclin 1 and LC3 autophagosomes (35, 61, 62). As decorin requires PEG3 for proper expression of Beclin 1 and LC3, we evaluated the role of TFEB in mediating Beclin 1 and LC3 induction following decorin treatment. Silencing TFEB not only prevented basal levels of *BECN1* (Fig. 3*F*) and *MAP1LC3A* (Fig. 3*G*) but severely impaired expression of both genes in response to decorin (Fig. 3, *F* and *G*).

Investigating Beclin 1 and LC3 protein levels revealed a similar pattern insofar as loss of TFEB abrogated Beclin 1 induction (Fig. 3*H*) but did not perturb the basal levels of Beclin 1 (Fig. 3*H*), in contrast to the loss of PEG3 (31). Further, in the presence of siScr, decorin promoted the conversion of LC3-I to LC3-II, but loss of TFEB stopped formation of the lipidated form (Fig. 2*H*). Interestingly, the basal levels of LC3-I appeared to be lower in the absence of TFEB (Fig. 3*H*).

Further implicating TFEB for decorin-evoked autophagosome formation, we performed differential interference contrast (DIC) microscopy on HUVECs. Immunostaining for Beclin 1 and LC3 in the presence of decorin led to the formation of dually positive autophagosomes (Fig. 3*I*). Conversely, transient depletion of TFEB prevented autophagosome formation following decorin application (Fig. 3*I*).

As TFEB has a role in the development of autophagosomes, we evaluated whether TFEB is required for autophagic flux. We transiently depleted TFEB and found substantially impaired flux for LC3 and p62/SQSTM1 (two established autophagic substrates) following decorin (Fig. 3*J*). Intriguingly, application of bafilomycin A1 alone appears to affect TFEB, suggesting that TFEB is sensitive to perturbations in basal flux. These data reinforce the concept of a common pathway involving TFEB for the induction of core autophagic genes, autophagosome formation, and flux in response to decorin.

### Decorin promotes TFEB nuclear translocation in a PEG3-dependent manner

**Figure 4.**
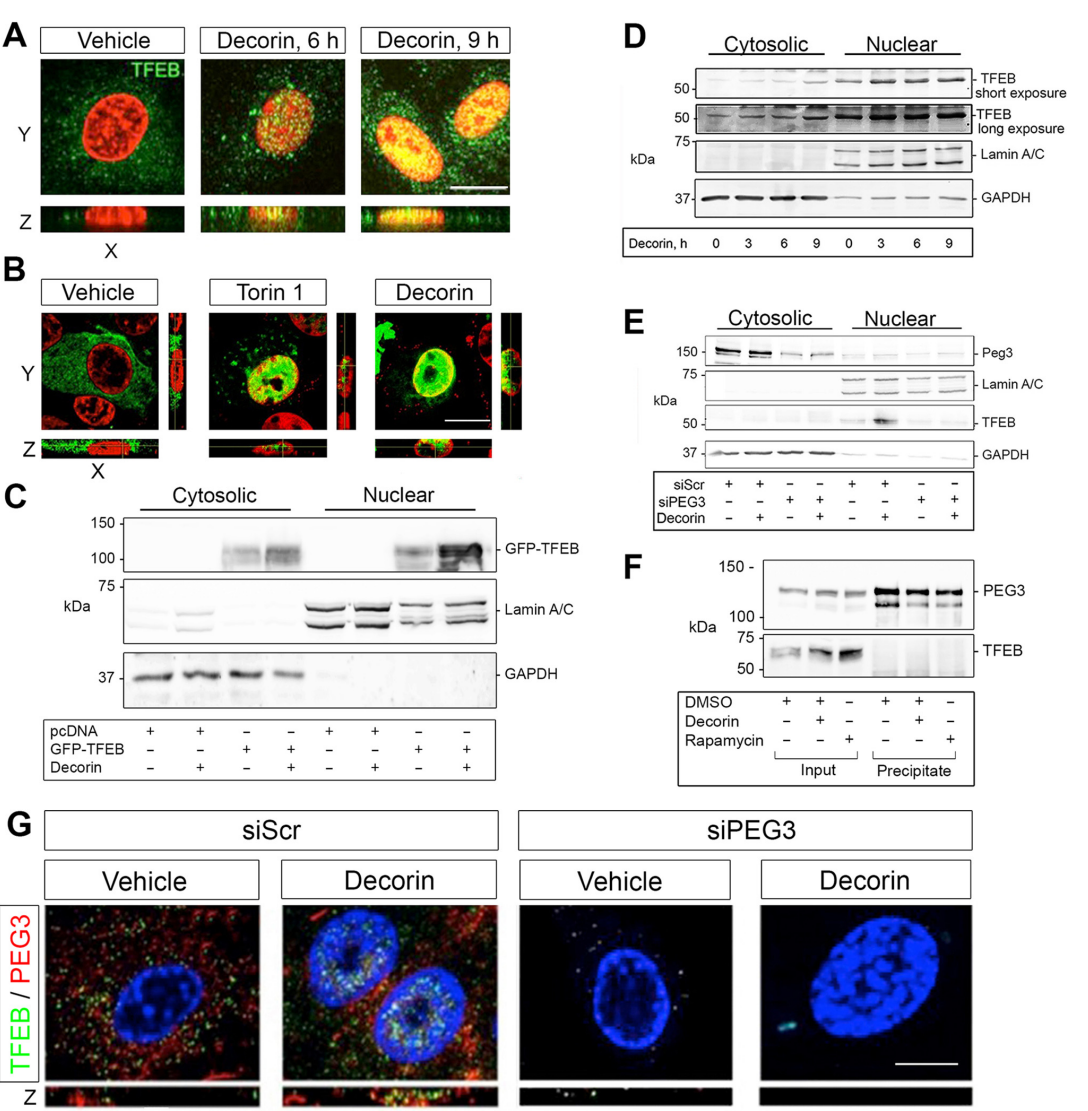
**Decorin promotes TFEB nuclear translocation in a PEG3-dependent manner.** *A*, confocal microscopy of TFEB localization in HUVECs following decorin. *B*, confocal microscopy of GFP-TFEB localization in PAER2 cells in the presence of Torin 1 (4 h, 20 μm) or decorin (6 h). *C*, cytosolic and nuclear fractionation of PAER2 cells transiently transfected with pcDNA or GFP-TFEB and stimulated with decorin (6 h). GFP-TFEB resolves around 100 kDa because of fusion with GFP at the N terminus. *D*, biochemical fractionation in HUVECs following decorin. *E*, biochemical fractionation in HUVECs following transfection with siScr or siPEG3 and stimulated with decorin. *F*, immunoprecipitation of transiently transfected PAER2 cells with HA-PEG3 and immunoblotted with anti-HA or TFEB following treatment with decorin or rapamycin. *G*, confocal microscopy of HUVECs. Nuclei (false-colored *red* in *A* and *B*) were visualized with DAPI. *Scale bar* = 10 μm. In *C*, *D*, and *E*, GAPDH and Lamin A/C were used to define the cytoplasmic and nuclear fractions, respectively. For confocal microcopy in *A*, *B*, and *G*, at least five fields/condition with z-stacks were captured for each of the three biological replicates in HUVECs or PAER2 cells. For the immunoblots in *C*, *D*, *E*, and *F*, data are representative of at least three independent biological replicates.

It is well-established that suppression of mTORC1 signaling results in TFEB translocation into the nucleus, where it induces the transcription of genes required for lysosomal biogenesis (49). Using confocal laser microscopy, we directly evaluated this process in response to decorin in HUVECs. Soluble decorin promoted nuclear accumulation of endogenous TFEB over time, with the appearance of yellow speckles at 6 h and broad nuclear co-localization at 9 h (Fig. 4*A*). Moreover, the TFEB signal increased concurrently, with a more punctate staining pattern within the cytosol (Fig. 4*A*).

To further validate these findings and to directly visualize movement of TFEB into the nucleus, we utilized an expression vector harboring TFEB fused at the N terminus to GFP (GFP-TFEB). As a positive control, we used Torin 1, a known autophagic inducer that specifically inhibits mTORC1 and mTORC2 (63). Both decorin and Torin 1 caused a marked translocation of TFEB into the nuclei of PAER2 cells (Fig. 4*B*). Next, we performed cytosolic and nuclear fractionation with PAER2 cells transiently transfected with either pcDNA or GFP-TFEB to recapitulate the confocal findings (Fig. 4*C*). We found that decorin augmented GFP-TFEB within the nuclear compartment over basal conditions. Moreover, decorin also increased GFP-TFEB within the cytoplasmic compartment, most likely as a result of mTOR suppression. We validated the morphological analyses via fractionation of HUVECs (Fig. 4*D*). Decorin promoted a progressive accumulation of TFEB in the nuclear fraction and a corresponding increase of TFEB signal in the cytosolic fraction (Fig. 4*D*). We included a longer exposure of the membrane immunoreacted for TFEB to clearly demonstrate the presence of TFEB under vehicle conditions within the cytoplasmic fraction.

Having shown a mechanistic dependence of TFEB induction on PEG3 activity, we next evaluated the role of PEG3 on the nuclear translocation of TFEB. Loss of PEG3 (Fig. 4*E*, cytosolic fraction) prevented decorin-evoked TFEB accumulation in the nuclei (Fig. 4*E*, nuclear fraction). Next, we determined whether TFEB nuclear translocation was due to an interaction of PEG3 with TFEB following autophagic stimulation. We transiently transfected PAER2 cells with HA-PEG3, immunoprecipitated PEG3, and evaluated TFEB binding following stimulation with decorin or rapamycin. We found that decorin and rapamycin increased TFEB levels within the inputs but did not result in any detectable interaction of PEG3 with TFEB (Fig. 4*F*). Thus, our data indicate that PEG3 does not bind TFEB for translocation and most likely utilizes an indirect pathway for nuclear translocation of TFEB (see below).

This result was morphologically verified, where decorin promoted an increase in PEG3 and TFEB (Fig. 4*G*) with accumulation of TFEB in the nucleus. However, following PEG3 silencing, basal TFEB protein levels decreased (Fig. 1*G*), precluding TFEB translocation (Fig. 4*G*).

Collectively, these data support a role for decorin in promoting the nuclear localization of TFEB. This effect is dependent upon Peg3 in maintaining basal TFEB levels for its consequent nuclear translocation in endothelial cells.

## Discussion

In this study, we report a novel, mechanistic link between a master regulator of autophagy, PEG3 (31), and TFEB, a master transcription factor required for lysosomal biogenesis and autophagy (48). Decorin, signaling via VEGFR2 and AMPK, transduces pro-autophagic cues for TFEB transcriptional induction and subsequent nuclear translocation in a strictly PEG3-dependent manner. These functions are biologically compatible with findings that posit decorin as a soluble, matrix-derived autophagic inducer necessary for endothelial cell autophagy (31), tumor cell mitophagy (64), and proper *in vivo* autophagic flux in cardiac muscle (65). Further, decorin expression appears to be transcriptionally up-regulated under conditions of organismal stress (*e.g.* sepsis (66) and starvation (65, 67)). Autophagic induction may underlie the core mechanisms that permit decorin to suppress tumorigenesis and angiogenesis (16). Intriguingly, these findings may also be applicable to TFE3, a TFEB homologue also responsive to autophagic stimuli (68).

Several insights into the mechanism of decorin-evoked endothelial cell autophagy can be deduced from these findings that may also be applicable to other pro-autophagic matrix molecules and proteoglycans (9). Unlike the requirement of PEG3 to maintain basal levels of both Beclin 1 mRNA and protein (31), it appears that PEG3 is recruited for TFEB expression only under induced conditions and not for maintaining basal *TFEB* levels. However, PEG3 loss does affect basal levels of TFEB protein, suggesting a post-translational role for conferring TFEB stability.

A similar role for TFEB has emerged in possibly orchestrating decorin-dependent conversion of LC3-I to lipidated LC3-II, perhaps via one of the CLEAR network targets TFEB is known to regulate (69). Alternately, TFEB has recently been linked to regulating cellular lipid metabolism via an autoregulatory loop (70), and, in conjunction with the known role of lipid regulation by autophagy (71), it is possible that the conversion defect of LC3 is rooted in abnormal lipid processing upon TFEB loss. Moreover, we have shown, for the first time, a role for TFEB in mediating the formation of Beclin 1/LC3-positive autophagosomes as well as maintaining autophagic flux downstream of external stimuli for autophagic induction within endothelial cells.

As decorin requires AMPK for efficient TFEB induction and nuclear localization, it is possible that a pathway involving AMPK/SKP2/CARM1 (54) might be engaged downstream of the decorin–VEGFR2–AMPK signaling axis. It would be of interest to evaluate, in future studies, the recruitment of CARM1, a histone arginine methyltransferase, to TFEB-positive transcriptional complexes at target promoters following decorin, as has been shown for traditional autophagic stimuli (54).

Collectively, we have identified a crucial downstream transcription factor for decorin-evoked autophagy that provides a more detailed understanding of the core processes operating during autophagic induction. Moreover, we have linked, for the first time, decorin to lysosomal homeostasis, a key facet of autophagy. In-depth investigations into the signaling pathways and elucidation of the primary components decorin utilizes will ultimately provide innovative and effective autophagy-based (73) therapeutic targets and solutions for human disease and cancer progression.

## Experimental procedures

### Cells, chemicals, and reagents

HUVECs were obtained from Lifeline Cell Technology (Frederick, MD), grown in basal medium supplemented with the VascuLife EnGS LifeFactors Kit (Lifeline Cell Technology), and used within the first five passages. Porcine aortic endothelial cells overexpressing VEGFR2 were described previously (74, 75). Rabbit polyclonal antibodies against TFEB, Lamin A/C, and GAPDH were obtained from Cell Signaling Technology. Rabbit antibody against Peg3 and goat polyclonal LC3 antibody were purchased from Santa Cruz Biotechnology (Santa Cruz, CA). Rabbit polyclonal anti-LC3B, Compound C, and SU5416 were purchased from Sigma. The rabbit-anti-Beclin 1 and HRP-conjugated goat anti-rabbit and donkey anti-mouse secondary antibodies were obtained from EMD Millipore (Billerica, MA). A custom rabbit polyclonal antibody (denoted P164) against the N-terminal human SCAN domain of Peg3 (spanning 14 amino acids from 164–177) was used for imaging. Torin 1 was purchased from Tocris (Bristol, UK). All primary antibodies were used at 1:1000 dilution in 1% BSA/TBST (Tris-buffered saline with Tween 20), except for GAPDH, which was used at 1:10,000. For immunofluorescence, primary antibodies were used at 1:200 in 1% BSA in PBS. Secondary antibodies for chemiluminescence were used at 1:5000 in the same buffer as above. SuperSignal West Pico enhanced chemiluminescence substrate was purchased from Thermo Fisher Scientific. Purification and validation of human recombinant decorin have been described elsewhere (22). Highly purified decorin proteoglycan (for purity, see Buraschi *et al.* (31)) was used at 200 nm throughout the study.

### Transient DNA and RNAi-mediated silencing

We transiently transfected PAER2 cells with increasing amounts of plasmid encoding HA-Peg3 using Lipofectamine 2000 (Life Technologies) in Opti-MEM (Gibco). Expression was verified by qPCR or immunoblotting where appropriate (see below). A full description of the DNA transfection protocol has been provided elsewhere (64). HUVECs were transiently transfected using Lipofectamine RNAiMAX (Life Technologies) mixed with siRNA against *H. sapiens PEG3* or *TFEB* mRNA (Santa Cruz Biotechnology). Scrambled siRNA (sc-37007, Santa Cruz Biotechnology) served as a control for all siRNA experiments presented here. The protocol for siRNA-mediated silencing is described elsewhere (55).

### Immunofluorescence and confocal laser microscopy

Typically, ∼5 × 10^4^ HUVECs were plated on 0.2% gelatin-coated 4-well chamber slides (Nunc, Thermo Scientific) and grown to full confluence in their growth media at 37 °C. Cells were subjected to immunofluorescence studies as described before (76, 77). Slides were incubated with conjugated secondary antibodies such as goat anti-rabbit IgG Alexa Fluor® 488 and goat anti-mouse IgG Alexa Fluor® 564 (Invitrogen). Nuclei were visualized with DAPI (Vector Laboratories).

Immunofluorescence images were acquired with a ×63, 1.3 oil immersion objective on a Leica DM5500B microscope equipped with the Leica application suite and advanced fluorescence v1.8 software (Leica Microsystems, Frankfurt, Germany). Confocal analyses were carried out utilizing a ×63, 1.3 oil immersion objective of a Zeiss LSM-780 confocal laser-scanning microscope with Zen imaging software. Images were captured as part of a z-stack series with 3-μm optical slices. A full description of the immunofluorescence and confocal laser microscopy protocol can be found elsewhere (31).

### Quantitative real-time PCR

Expression analysis by quantitative real-time PCR (qPCR) was carried out on subconfluent 6-well plates seeded with ∼2 × 10^5^ HUVECs or PAER2 cells and harvested in TRIzol reagent (Invitrogen) following the appropriate experimental conditions. Gene expression analysis was performed on a Roche LightCycler 480-II and calculated with the comparative Ct (thermal cycle) method. A full description can be found in Refs. 31, 72.

### Cytosolic and nuclear fractionation

Approximately 2 × 10^5^ HUVECs were seeded and treated according to the experimental conditions. NE-PER nuclear and cytoplasmic extraction reagents (Thermo Fisher) were used for fractionation according to the instructions of the manufacturer.

### Quantification and statistical analysis

Immunoblots were quantified by scanning densitometry using Scion Image software (National Institutes of Health). Graphs were generated using Sigma Stat 3.10. Experiments with three or more comparison groups were subjected to one-way ANOVA followed by a Bonferroni post hoc test. Differences among the conditions were considered significant at *p* < 0.05.

## Author contributions

R. V. I. and T. N. designed the study, analyzed the data, and wrote the manuscript. T. N. and C. S. performed the research. R. T. O. provided valuable research reagents.

## Footnotes

3 The abbreviations used are:

PEG: paternally expressed gene
VEGFR: VEGF receptor
AMPK: AMP-activated protein kinase
mTOR: mammalian target of rapamycin
TFEB: transcription factor EB
HUVEC: human umbilical vein endothelial cell
qPCR: quantitative real-time PCR
ZF: zinc finger
DIC: differential interference contrast
ANOVA: analysis of variance
siScr: scramble siRNA.
